# Clinical characteristics and pharmacovigilance signals of immune checkpoint inhibitor-associated cystitis and ureteritis: analysis of FAERS reports and published cases

**DOI:** 10.3389/fphar.2026.1918548

**Published:** 2026-10-16

**Authors:** Yuanyuan Zhang, Jiajin Yang

**Affiliations:** 1 Department of Pathology, Fengcheng People’s Hospital, Yichun University, Yichun, Jiangxi, China; 2 Department of Oncology, Fengcheng People’s Hospital, Yichun University, Yichun, Jiangxi, China

**Keywords:** cystitis, FAERS, immune checkpoint inhibitors, immune-related adverse events, pharmacovigilance, ureteritis

## Abstract

**Background:**

Immune checkpoint inhibitor (ICI)-associated cystitis and ureteritis are uncommon urinary immune-related adverse events that are difficult to distinguish from infection or other urinary tract complications. Their clinical phenotype and pharmacovigilance signal remain incompletely defined.

**Methods:**

We combined a pooled analysis of published cases with a FAERS disproportionality analysis from 2004Q1 to 2026Q1. Published cases were reviewed for clinical presentation, treatment, rechallenge, and time-to-onset. FAERS reports were deduplicated at the CASEID level. A broad cystitis-inclusive definition included IMMUNE-MEDIATED CYSTITIS, CYSTITIS NONINFECTIVE, URETERITIS, and CYSTITIS; a high-specificity definition excluded the nonspecific Preferred Term CYSTITIS. Disproportionality was assessed using reporting odds ratios (RORs), proportional reporting ratios, chi-square statistics, and information components.

**Results:**

Twenty-eight reports comprising 45 unique patients were included. The median age was 56 years, and lung cancer was the most common malignancy. Published cases commonly showed urinary frequency, dysuria, hematuria, leukocyturia, inflammatory imaging or cystoscopic abnormalities, and corticosteroid responsiveness. Upper urinary tract involvement was documented in 13 of 33 patients with available CT data. Median time-to-onset was 62 days among 24 evaluable cases, and recurrence occurred in 5 of 9 rechallenged patients. In FAERS, 20,257,224 deduplicated CASEIDs were analyzed. The broad cystitis-inclusive definition identified 397 ICI-associated cases and showed only a weak signal (ROR, 1.12; 95% CI, 1.02–1.24), whereas the high-specificity definition identified 151 cases and yielded a stronger signal (ROR, 9.04; 95% CI, 7.63–10.70). Class-level signals were also observed, and the overall high-specificity signal remained positive across sensitivity analyses.

**Conclusion:**

These findings support ICI-associated cystitis and ureteritis as an uncommon urothelial inflammatory irAE spectrum. Phenotype-aware outcome definition is important in pharmacovigilance because nonspecific cystitis coding may dilute the reporting signal.

## 1 Introduction

Immune checkpoint inhibitors (ICIs) restore antitumor immunity by blocking inhibitory pathways such as programmed cell death protein 1 (PD-1), programmed death-ligand 1 (PD-L1), and cytotoxic T-lymphocyte-associated protein 4 (CTLA-4). Their use has expanded across solid tumors, *including genitourinary malignancies such as urothelial carcinoma* (Liu et al., 2026; Song et al., 2025; Song et al., 2024)*,* but the same immune activation can disrupt peripheral tolerance and produce immune-related adverse events (irAEs) across multiple organs (Schneider et al., 2021; Brahmer et al., 2021; Haanen et al., 2022; Thompson et al., 2024; Yin et al., 2023). The organ distribution, timing, and severity of irAEs vary across ICI classes and treatment regimens, making toxicity recognition dependent on both clinical context and exclusion of competing diagnoses (Wang et al., 2025; Li et al., 2022; Inoue and Inui, 2026). Accurate phenotyping is therefore central to separating immune toxicity from cancer progression, infection, treatment-related tissue injury, and unrelated comorbidity (Wang et al., 2025; Li et al., 2022; Zhu L. et al., 2021).

Urinary tract irAEs are uncommon within this spectrum (Wang et al., 2025; Frey, 2026). The first published case of nivolumab-associated cystitis described sterile urinary symptoms, diffuse bladder mucosal inflammation, biopsy-based exclusion of malignancy, improvement with corticosteroids, and successful nivolumab resumption under concomitant steroid therapy (Ozaki et al., 2017). Subsequent reports broadened the phenotype from isolated cystitis to ureteritis and cystitis involving the upper urinary tract, with sterile pyuria, hematuria, hydroureteronephrosis, ureteral or bladder wall thickening, and occasional kidney dysfunction (Wang et al., 2025; Li et al., 2022; Tu et al., 2021). These manifestations overlap with bacterial urinary tract infection, tumor involvement, urinary obstruction, radiation injury, and drug-induced cystitis from other agents (Zhu L. et al., 2021; Ueki et al., 2020). Consequently, patients may receive repeated antibiotics before an immune-mediated diagnosis is considered (Yuan et al., 2025).

The published evidence has largely consisted of case reports and small case series, with variation in terminology, diagnostic evaluation, corticosteroid dose, and ICI rechallenge (Wang et al., 2025; Fukunaga et al., 2022). A recent single-center cohort described 12 patients with ICI-related ureteritis and cystitis and highlighted upper urinary tract involvement and relapse during corticosteroid tapering (Wang et al., 2025). However, broader patient-level synthesis and outcome-sensitive pharmacovigilance evaluation remain limited.

Spontaneous-reporting data offer a complementary population-scale perspective but introduce a distinct methodological problem (Frey, 2026). The MedDRA Preferred Term “CYSTITIS” is etiologically broad and may capture infectious, radiation-related, malignant, or otherwise unspecified events. Combining this term with more specific noninfective, immune-mediated, and ureteritis terms may therefore dilute or obscure an ICI-associated reporting pattern (Schotland et al., 2018). We therefore conducted a two-part study combining a patient-level pooled analysis of published cases with a CASEID-deduplicated FAERS analysis from 2004Q1 through 2026Q1. The objective was to characterize published clinical cases and evaluate pharmacovigilance signals of ICI-associated cystitis and ureteritis using FAERS, with separate broad cystitis-inclusive and phenotype-enriched high-specificity MedDRA outcome definitions.

## 2 Methods

### 2.1 Study design and data sources

We conducted a two-part study combining a pooled analysis of published cases with a disproportionality analysis of the FDA Adverse Event Reporting System (FAERS). The design, reporting, and interpretation of the disproportionality analysis followed key READUS-PV principles (Fusaroli et al., 2024a; Fusaroli et al., 2024b). Published cases were used to characterize clinical presentation, treatment, recurrence after rechallenge, and time-to-onset (TTO). FAERS data were used to evaluate reporting signals and describe high-specificity reports involving ICI-associated cystitis or ureteritis.

FAERS quarterly ASCII files from 2004Q1 through 2026Q1 were downloaded and analyzed at the CASEID level. Duplicate or follow-up reports were consolidated by retaining the latest version for each CASEID according to FDA_DT. When multiple reports shared the same FDA_DT, the report with the numerically largest PRIMARYID or ISR was retained.

### 2.2 Literature search and published-case extraction

By June 2026, we performed a structured literature review to identify published individual cases. *Because available evidence consisted predominantly of case reports and small case series, this review was designed for phenotype characterization rather than a systematic review with quantitative synthesis.* Search terms combined ICI-related terms with urinary tract toxicity terms, including immune checkpoint inhibitor, PD-1, PD-L1, CTLA-4, nivolumab, pembrolizumab, atezolizumab, durvalumab, ipilimumab, cystitis, non-bacterial cystitis, immune-mediated cystitis, ureteritis, and cystoureteritis. Reference lists of eligible articles and relevant reviews were also screened.

Eligible publications described individual patients who developed cystitis, noninfectious cystitis, immune-mediated cystitis, ureteritis, or cystoureteritis during or after ICI treatment. Reports were excluded when the urinary tract event was clearly attributed to bacterial infection, radiation injury, tumor invasion, urinary obstruction, or another non-ICI cause. When overlap was suspected, patient-level details were compared, and duplicate patients were counted once.

Extracted variables included demographics, cancer type, ICI regimen, cycles before onset, TTO, urinary manifestations, urinalysis and culture findings, imaging and cystoscopic findings, pathology, treatment, urinary outcome, death during follow-up, and recurrence after ICI rechallenge. Published-case TTO was defined as the interval from ICI initiation to symptom onset.

### 2.3 FAERS outcome definitions

Two outcome definitions were used. The broad definition included IMMUNE-MEDIATED CYSTITIS, CYSTITIS NONINFECTIVE, URETERITIS, and CYSTITIS. Because CYSTITIS is etiologically nonspecific, a high-specificity definition excluding this term was also used. The high-specificity definition included IMMUNE-MEDIATED CYSTITIS, CYSTITIS NONINFECTIVE, and URETERITIS.

The phenotype-enriched high-specificity definition excluded the nonspecific PT CYSTITIS and was used for the main interpretation. This definition was considered phenotype-enriched rather than immune-confirmed, because MedDRA coding cannot establish immune attribution at the individual-report level.

### 2.4 ICI exposure definition

The ICI exposure set included nivolumab, pembrolizumab, cemiplimab, dostarlimab, retifanlimab, toripalimab, sintilimab, camrelizumab, tislelizumab, atezolizumab, durvalumab, avelumab, ipilimumab, tremelimumab, and cadonilimab. Drug names were standardized before matching. ICI exposure was defined as an eligible ICI coded as primary suspect (PS) or secondary suspect (SS) in the FAERS DRUG table.

Agents were grouped as PD-1 inhibitors, PD-L1 inhibitors, and CTLA-4 inhibitors. Class-level and agent-level analyses were not mutually exclusive because a single CASEID could include sequential or combination ICI exposure.

### 2.5 Disproportionality analysis

Disproportionality analyses were performed using 2 × 2 contingency tables at the deduplicated CASEID level (Bate and Evans, 2009). For each exposure group, the table was defined as follows: a, ICI-exposed CASEIDs with the outcome; b, ICI-exposed CASEIDs with all other adverse events; c, non-ICI CASEIDs with the outcome; and d, non-ICI CASEIDs with all other adverse events. Reporting odds ratios (RORs) with 95% confidence intervals, proportional reporting ratios (PRRs), Pearson chi-square statistics, and Bayesian information components (ICs) with IC025 values were calculated. IC estimates used a continuity-corrected Bayesian shrinkage approach.

A positive ROR signal was defined as at least three cases and a lower 95% confidence limit greater than 1. A positive PRR signal required at least three cases, PRR ≥2, and chi-square ≥4. A positive Bayesian signal was defined as IC025 > 0.

Continuous variables were summarized as medians with interquartile ranges, and categorical variables as counts and percentages. Analyses were performed using R software.

### 2.6 Sensitivity analyses

Three sensitivity analyses were conducted. First, ICI exposure was restricted to drugs coded as primary suspect. Second, CASEIDs with co-reported urinary infection-related Preferred Terms were excluded, including urinary tract infection, pyelonephritis, urosepsis, bacteriuria, pyuria, kidney infection, bladder infection, and explicitly infectious cystitis terms. Third, the FAERS background population was restricted to reports from 2014Q1 through 2026Q1.

### 2.7 Descriptive analyses and exploratory FAERS time-to-onset

For the 151 ICI-associated high-specificity FAERS cases, demographic characteristics, reporting year, seriousness outcomes, cancer indications, ICI class, ICI agent, and Preferred Term composition were summarized descriptively.

FAERS-based TTO was analyzed only when both ICI start date and event onset date were complete, interpretable, and yielded a nonnegative interval. ICI start dates were derived from START_DT in the THER table and matched to eligible ICI drugs using drug sequence identifiers. Event onset dates were derived from EVENT_DT in the DEMO table. FDA_DT was not used as a substitute for onset date. Because evaluable dates were available in only a minority of reports, FAERS TTO was treated as exploratory.

## 3 Results

### 3.1 Published-case selection and clinical characteristics

A total of 28 full-text reports comprising 45 unique patients with ICI-associated cystitis or ureteritis were included in the pooled published-case analysis (Figure 1A). The median age was 56 years (IQR, 49.5–64.5; range, 27–78), and 30 patients were male. Lung cancer was the most common malignancy (16/45, 35.6%), followed by gastric cancer (11/45, 24.4%). PD-1 inhibitors were the predominant ICI class (41/45, 91.1%); the most common individual agents were nivolumab (14/45, 31.1%), pembrolizumab (12/45, 26.7%), and sintilimab (9/45, 20.0%) (Table 1) (Schneider et al., 2021; Wang et al., 2025; Li et al., 2022; Zhu L. et al., 2021; Ozaki et al., 2017; Tu et al., 2021; Ueki et al., 2020; Yuan et al., 2025; Fukunaga et al., 2022; El Husseini et al., 2021; Li and Lin, 2026; Peng et al., 2026; Zhu S. et al., 2021; Alhusari et al., 2024; Zhang et al., 2024; Obayashi et al., 2022; Ji et al., 2024; Zhou et al., 2023; Yan et al., 2026; Yajima et al., 2021; Ni et al., 2023; Shimatani et al., 2018; Di et al., 2024; He et al., 2022; Li et al., 2025; Fan et al., 2023; Wang et al., 2023; Anraku et al., 2023).

**FIGURE 1:**
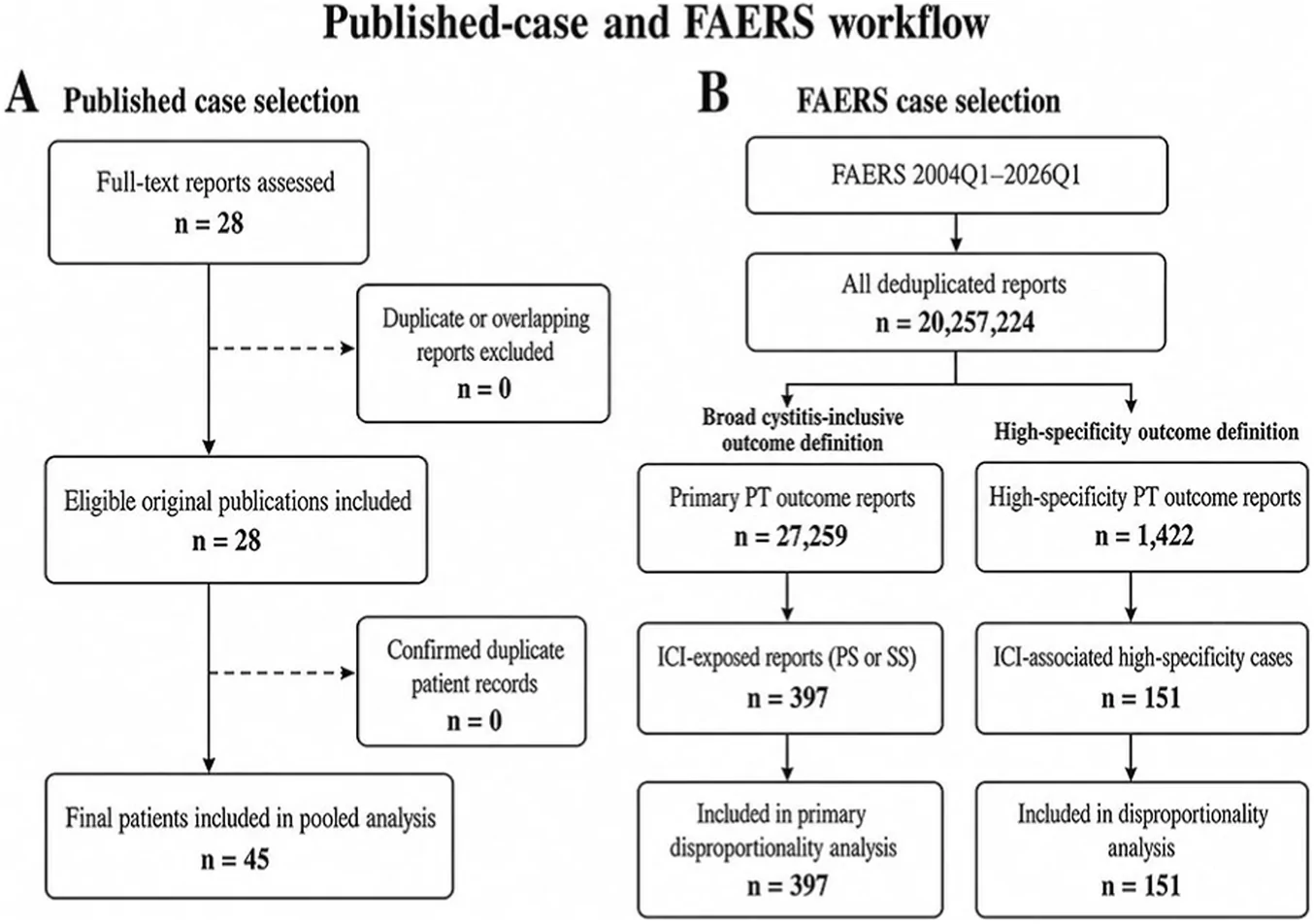
Workflow of published-case selection and FAERS case identification. **(A)** Selection of published cases included in the pooled patient-level analysis. **(B)** Identification of FAERS reports included in the disproportionality analysis. FAERS, FDA Adverse Event Reporting System; ICI, immune checkpoint inhibitor; PT, Preferred Term; PS, primary suspect; SS, secondary suspect.

**TABLE 1:** Clinical characteristics of published cases.

Characteristic	Value
Total patients	45
Age, years, median (IQR; range), n = 31	56 (49.5–64.5; 27–78)
Sex, male/female/not reported	30/13/2
Most common cancers	Lung cancer 16/45 (35.6%); gastric cancer 11/45 (24.4%)
Other cancers	18/45 (40.0%)
ICI class exposure	PD-1 inhibitors 41/45 (91.1%); PD-L1 inhibitors 3/45 (6.7%); CTLA-4 inhibitors 3/45 (6.7%); bispecific antibody 2/45 (4.4%)
Most common ICI agents	Nivolumab 14/45 (31.1%); pembrolizumab 12/45 (26.7%); sintilimab 9/45 (20.0%)
Time to onset, days, median (IQR; range), n = 24	62 (21–141; 7–442)
Common urinary findings	Frequency 40/41 (97.6%); dysuria 30/31 (96.8%); hematuria 31/31 (100.0%); leukocyturia 42/43 (97.7%)
Imaging and cystoscopy findings	Abnormal CT 31/33 (93.9%); upper urinary tract involvement 13/33 (39.4%); abnormal cystoscopy 19/20 (95.0%)
Pathological findings	Inflammatory pathology 15/18 (83.3%); T-cell/CD8-predominant infiltration 8/18 (44.4%)
Corticosteroid treatment	37/45 (82.2%)
Second-line immunomodulation	3/45 (6.7%)
Urinary drainage, stent, or nephrostomy	3/45 (6.7%)
ICI rechallenge performed	9/45 (20.0%)
Recurrence after rechallenge	5/9 (55.6%)
Urinary recovery or improvement	45/45 (100.0%)
All-cause death during follow-up	7/45 (15.6%)

ICI, immune checkpoint inhibitor; IQR, interquartile range; PD-1, programmed cell death protein 1; PD-L1, programmed death-ligand 1; CTLA-4, cytotoxic T-lymphocyte-associated protein 4; CT, computed tomography; CD8, cluster of differentiation 8.

The published cases showed a consistent inflammatory urinary phenotype. Frequency, dysuria, hematuria, and leukocyturia were reported in most evaluable patients. CT abnormalities were present in 31 of 33 patients (93.9%), including upper urinary tract involvement in 13 of 33 (39.4%). Cystoscopy was abnormal in 19 of 20 patients (95.0%). Inflammatory pathology was reported in 15 of 18 patients (83.3%), including T-cell or CD8-predominant infiltration in 8 of 18 (44.4%).

Time-to-onset was available in 24 published cases. The median interval from ICI initiation to cystitis or ureteritis onset was 62 days (IQR, 21–141; range, 7–442). Corticosteroids were used in 37 of 45 patients (82.2%). ICI rechallenge was performed in 9 patients, with recurrence in 5 of 9 (55.6%). Urinary improvement was reported in all published cases with available follow-up. TTO and treatment-related outcomes are summarized in Figure 2.

**FIGURE 2:**
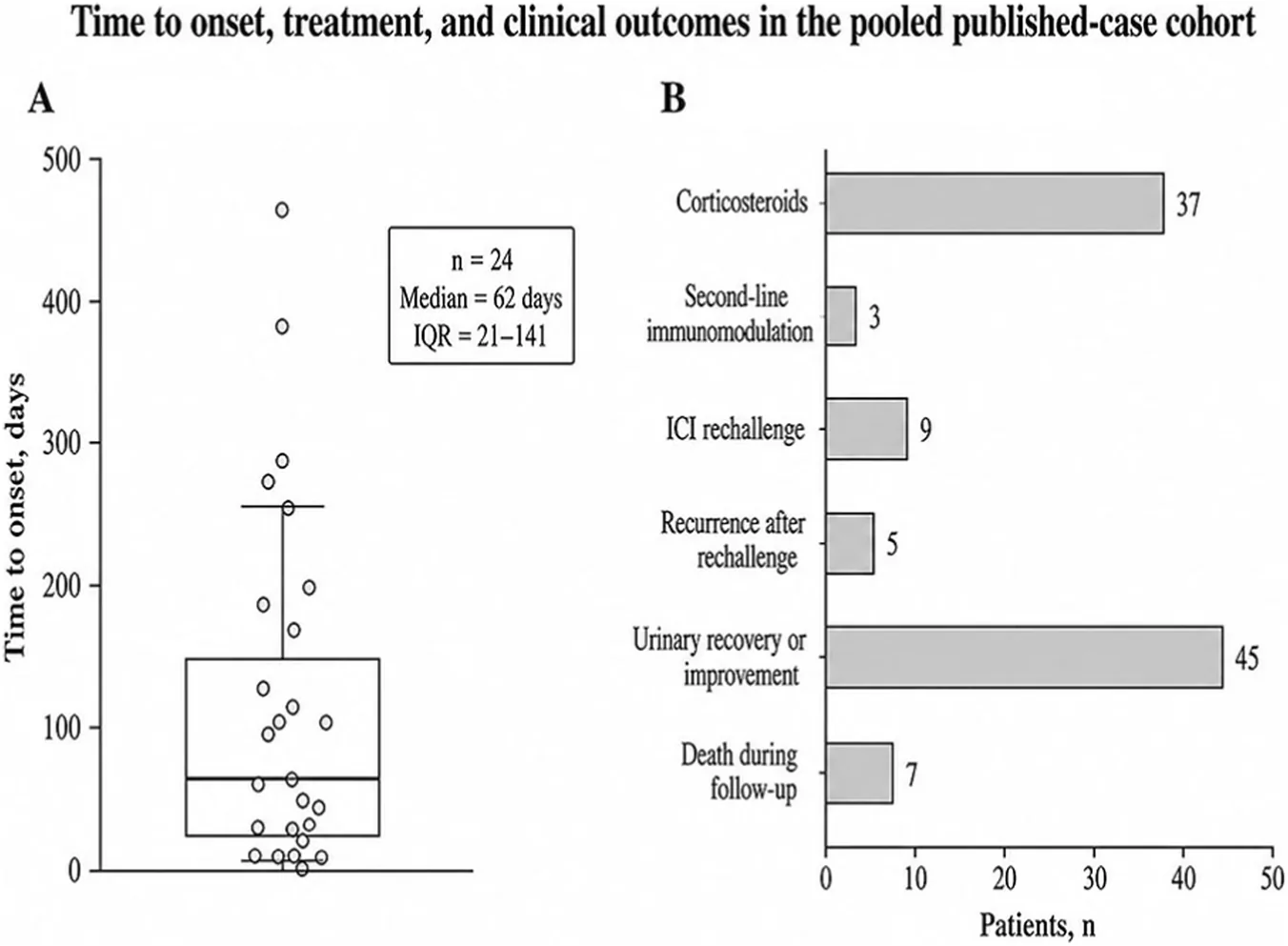
Time to onset, treatment, and clinical outcomes in the pooled published-case cohort. **(A)** Time-to-onset distribution among patients with evaluable onset intervals. **(B)** Summary of treatment and clinical outcomes. Second-line immunomodulation included infliximab and JAK inhibitor therapy; death during follow-up may overlap with urinary recovery. ICI, immune checkpoint inhibitor; IQR, interquartile range.

### 3.2 FAERS report selection and outcome-definition comparison

The FAERS source population comprised 20,257,224 deduplicated CASEIDs from 2004Q1 through 2026Q1. The broad cystitis-inclusive definition identified 27,259 outcome reports, of which 397 involved an eligible ICI coded as a primary or secondary suspect drug. The high-specificity outcome definition identified 1,422 outcome reports, including 151 ICI-associated high-specificity cases for the main disproportionality analysis (Figure 1B).

The broad cystitis-inclusive definition produced only a weak overall ICI signal, with 397 cases and an ROR of 1.12 (95% CI, 1.02–1.24). In contrast, the high-specificity definition yielded a markedly stronger signal, with 151 cases and an ROR of 9.04 (95% CI, 7.63–10.70) (Table 2).

**TABLE 2:** Disproportionality analysis of ICI-associated cystitis and ureteritis in FAERS.

Outcome/exposure	Cases, n	ROR (95% CI)	PRR	χ^2^	IC (IC025–IC975)	Signal
Broad cystitis-inclusive definition, overall ICIs	397	1.12 (1.02–1.24)	1.12	5.20	0.17 (0.03–0.31)	Weak/attenuated signal
High-specificity outcome definition, overall ICIs	151	9.04 (7.63–10.70)	9.03	964.00	3.04 (2.79–3.28)	Positive signal
PD-1 inhibitors	132	10.52 (8.79–12.58)	10.51	1030.58	3.27 (3.01–3.53)	Positive signal
PD-L1 inhibitors	16	3.98 (2.43–6.51)	3.98	35.27	2.02 (1.32–2.72)	Positive signal
CTLA-4 inhibitors	32	10.69 (7.53–15.18)	10.69	274.63	3.41 (2.91–3.91)	Positive signal
Nivolumab	55	8.89 (6.79–11.65)	8.89	370.27	3.11 (2.73–3.50)	Positive signal
Pembrolizumab	66	10.81 (8.44–13.84)	10.80	559.72	3.38 (3.03–3.74)	Positive signal
Ipilimumab	31	11.33 (7.94–16.18)	11.32	285.45	3.49 (2.98–4.00)	Positive signal
Atezolizumab	12	4.79 (2.71–8.46)	4.79	35.69	2.31 (1.50–3.11)	Positive signal

Broad cystitis-inclusive definition: IMMUNE-MEDIATED CYSTITIS; CYSTITIS NONINFECTIVE; URETERITIS; CYSTITIS., High-specificity outcome definition: IMMUNE-MEDIATED CYSTITIS; CYSTITIS NONINFECTIVE; URETERITIS., Class-level and agent-level rows used the high-specificity definition and PS/SS ICI, exposure. FAERS, FDA, adverse event reporting system; ICI, immune checkpoint inhibitor; IC, information component; PRR, proportional reporting ratio; ROR, reporting odds ratio.

### 3.3 Main high-specificity FAERS disproportionality signals

Using the high-specificity outcome definition, overall ICIs showed positive disproportionality across ROR, PRR, and Bayesian criteria, with an ROR of 9.04 (95% CI, 7.63–10.70), PRR of 9.03, and IC of 3.04 (IC025–IC975, 2.79–3.28). Positive signals were observed across PD-1, PD-L1, and CTLA-4 inhibitor classes. At the agent level, signals were observed for nivolumab, pembrolizumab, ipilimumab, and atezolizumab, with the highest point estimates observed for ipilimumab and pembrolizumab. Detailed estimates are shown in Table 2 and Figure 3.

**FIGURE 3:**
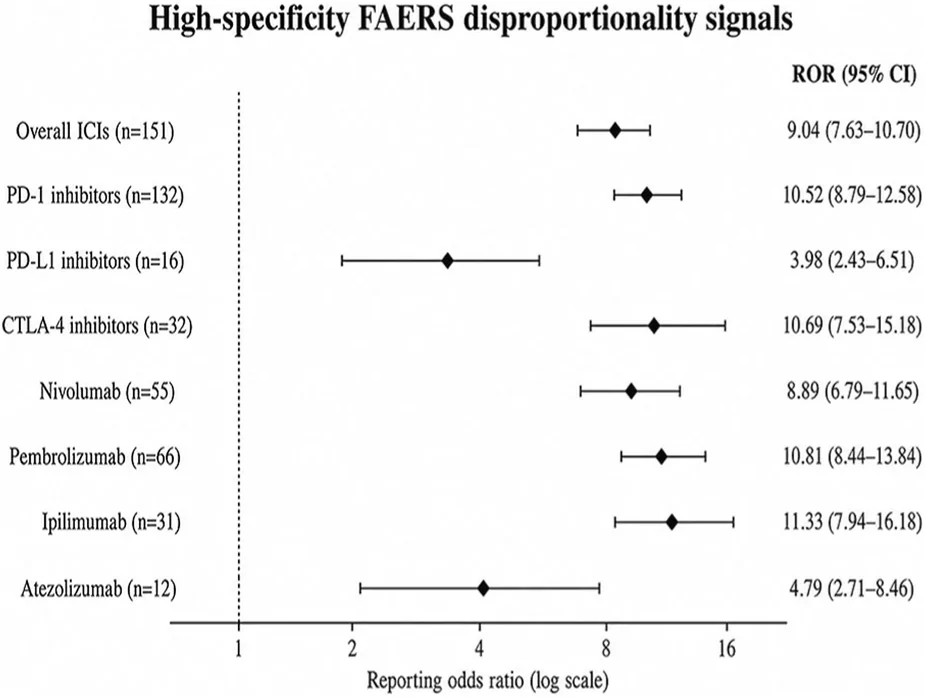
High-specificity FAERS disproportionality signals for ICI-associated cystitis and ureteritis. Forest plot of reporting odds ratios and 95% confidence intervals under the high-specificity outcome definition. The vertical reference line indicates an ROR of 1. FAERS, FDA Adverse Event Reporting System; ICI, immune checkpoint inhibitor; ROR, reporting odds ratio; CI, confidence interval.

### 3.4 Preferred Term composition

Among the 151 ICI-associated high-specificity cases, IMMUNE-MEDIATED CYSTITIS was the most frequent Preferred Term (96 cases, 63.6%), followed by CYSTITIS NONINFECTIVE (43 cases, 28.5%) and URETERITIS (19 cases, 12.6%). Seven cases (4.6%) involved overlapping high-specificity PTs (Supplementary Table S2).

### 3.5 Sensitivity analyses

The high-specificity signal remained stable across sensitivity analyses. In the PS-only analysis, overall ICIs accounted for 135 cases and had an ROR of 10.39 (95% CI, 8.70–12.41). After exclusion of 7 cases (4.6%) with co-reported urinary infection-related Preferred Terms, the overall signal remained positive, with 144 cases and an ROR of 10.67 (95% CI, 8.97–12.71). In the temporal sensitivity analysis restricted to 2014Q1 through 2026Q1, all 151 ICI-associated high-specificity cases were retained, and the overall ROR was 7.84 (95% CI, 6.62–9.29) (Supplementary Tables S3–S5).

### 3.6 Descriptive characteristics and reporting trend of FAERS high-specificity cases

Among the 151 ICI-associated high-specificity FAERS cases, age was available in 126 cases (83.4%), with a median of 61 years (IQR, 51.2–70). Male patients accounted for 79 cases (52.3%), sex was missing or unknown in 18 cases (11.9%). Serious outcomes were recorded in 148 cases (98.0%), most commonly hospitalization (65 cases, 43.0%); death was reported in 11 cases (7.3%). PD-1 inhibitors were the most frequently reported ICI class (132 cases, 87.4%), pembrolizumab was the most common individual agent (66 cases, 43.7%). Lung cancer was the most common indication group (39 cases, 25.8%) (Supplementary Table S6).

Annual reporting increased after 2021 and peaked in 2023, with 38 reports. No ICI-associated high-specificity reports were identified from 2004 through 2013, and 17 reports were recorded in 2026Q1 only, but 2026 data were incomplete and were not directly comparable with full-year counts (Figure 4).

**FIGURE 4:**
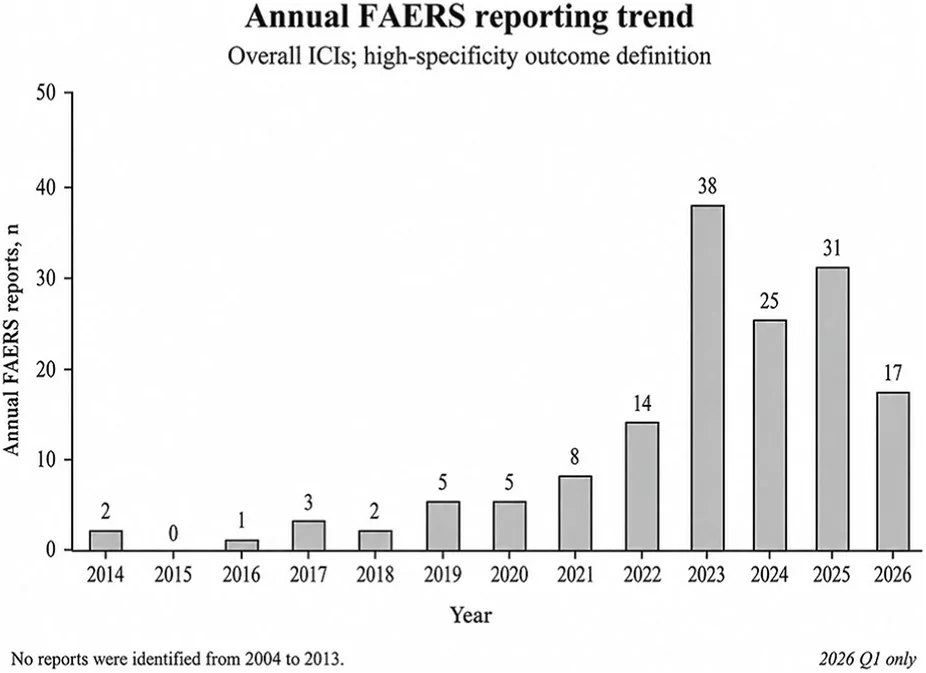
Annual reporting trend of ICI-associated cystitis and ureteritis in FAERS. Annual counts of CASEID-deduplicated FAERS reports for overall ICI exposure under the high-specificity outcome definition. No eligible reports were identified from 2004 to 2013; 2026 includes first-quarter data only. FAERS, FDA Adverse Event Reporting System; ICI, immune checkpoint inhibitor.

### 3.7 Exploratory FAERS time-to-onset analysis

In the exploratory FAERS TTO analysis, only 29 of 151 ICI-associated high-specificity cases (19.2%) had complete and interpretable treatment start and event onset dates. The median FAERS-based TTO was 36 days (IQR, 14–121; range, 0–1196) (Supplementary Table S7). Because most FAERS reports lacked evaluable dates, this analysis was considered supplementary and was not used as the primary TTO estimate.

## 4 Discussion

### 4.1 A urothelial inflammatory spectrum and diagnostic attribution

The findings of this study support ICI-associated cystitis and ureteritis as a broader urothelial inflammatory spectrum rather than a bladder-confined toxicity. These observations indicate that inflammatory involvement is not confined to the bladder and may extend across multiple segments of the urothelial tract (Zhu L. et al., 2021; Frey, 2026). This interpretation is supported by our pooled analysis, in which upper urinary tract involvement was documented in 13 of 33 patients with available CT imaging. These observations argue against treating cystitis and ureteritis as unrelated entities and instead support a phenotype-based view of immune-mediated urothelial injury.

The main clinical challenge is attribution. Dysuria, urgency, hematuria, pyuria, flank pain, and hydronephrosis are common in oncology practice and may reflect infection, stones, radiation injury, obstruction, catheter-related irritation, or tumor involvement. Across published cases, the more informative pattern was persistence of urinary symptoms despite antimicrobial therapy, repeatedly negative cultures or sterile pyuria, temporal association with ICI exposure, inflammatory imaging or cystoscopic abnormalities, and response to immunosuppression. Diagnosis should therefore rely on convergence of clinical, microbiological, radiological, endoscopic, pathological, and treatment-response evidence after competing causes have been excluded (Zhu L. et al., 2021; Frey, 2026; Fukunaga et al., 2022).

### 4.2 Pathological evidence and biological plausibility

Pathological findings provide biological support for immune-mediated urothelial injury, although available data remain limited (Zhu L. et al., 2021; Ueki et al., 2020; Di et al., 2024). Reported cases frequently showed inflammatory changes, and several demonstrated CD8-positive or T-cell-predominant infiltration of the urothelium and lamina propria (Zhu L. et al., 2021; Ueki et al., 2020; Di et al., 2024). *However, CD8-positive infiltration was not consistently reported among biopsied cases, and the available pathological descriptions were heterogeneous and insufficient to define a uniform histological subtype.* These findings are biologically plausible given the mechanism of action of PD-1/PD-L1 and CTLA-4 blockade, which enhance effector T-cell activity by removing inhibitory immune checkpoints (Wang et al., 2020). Interestingly, PD-L1 expression has been reported inconsistently. Some cases demonstrated strong urothelial PD-L1 staining, whereas others showed minimal or absent expression despite clear clinical evidence of immune-mediated disease (Zhu L. et al., 2021; Ueki et al., 2020; Obayashi et al., 2022). These observations suggest that urothelial PD-L1 expression alone may not reliably define disease activity or severity. Overall, the available pathology supports T-cell-mediated inflammation as a plausible mechanism, without establishing a single diagnostic histological pattern. *The lower reporting signal observed with PD-L1 inhibitors may have biological plausibility, because PD-1 blockade affects both PD-1/PD-L1 and PD-1/PD-L2 pathways, whereas PD-L1 blockade preserves PD-1/PD-L2 signaling. However, this interpretation remains speculative because FAERS cannot distinguish biological mechanisms from differences in clinical use or reporting behavior.*

### 4.3 Treatment response, relapse, and ICI rechallenge

Most reported patients improved rapidly after corticosteroid administration, supporting the concept that immune activation is a central driver of urinary tract injury (Wang et al., 2025; Li et al., 2022; El Husseini et al., 2021). However, the clinical course observed across published cases suggests that symptom resolution does not necessarily indicate restoration of immune tolerance (Wang et al., 2025; Fukunaga et al., 2022; Yan et al., 2026). Current irAE guidelines provide general principles for corticosteroid use, ICI interruption, and ICI rechallenge, but urinary tract irAEs are not addressed with the same granularity as more common toxicities (Schneider et al., 2021; Brahmer et al., 2021; Thompson et al., 2024).

The Wang cohort highlighted a frequently overlooked aspect of urinary irAEs: relapse during corticosteroid tapering. More than half of steroid-treated patients experienced symptom recurrence while immunosuppression was being reduced (Wang et al., 2025). Similar patterns were observed in individual case reports, where prolonged corticosteroid exposure, second-line immunomodulation, or repeated treatment courses were required to maintain disease control (Fukunaga et al., 2022; Li and Lin, 2026; Yan et al., 2026). These findings suggest that the underlying immune process may persist beyond the initial inflammatory episode in a subset of patients.

The rechallenge experience further supports this interpretation. In our pooled analysis, recurrence occurred in 5 of 9 rechallenged patients (55.6%). Interestingly, the recurrence rate observed after rechallenge (55.6%) was comparable to the relapse frequency reported during corticosteroid tapering in the Wang cohort (60%). *Although the numerical frequencies were similar, these two events likely represent different clinical processes. Rechallenge recurrence may reflect renewed immune activation after antigen exposure, whereas relapse during corticosteroid tapering may indicate incomplete resolution of the initial inflammatory response. Therefore, the comparable rates should be interpreted as a shared observation of disease reactivation rather than evidence of identical immunological mechanisms* (Wang et al., 2025; Ozaki et al., 2017; Peng et al., 2026; Fan et al., 2023). The report by Fan et al., in which cystitis recurred after sequential exposure to two different PD-1 inhibitors, further suggests that recurrence may reflect broader immune susceptibility rather than a reaction restricted to a single agent (Fan et al., 2023).

Taken together, these data indicate that urinary irAEs are not invariably self-limited. The evidence remains insufficient to define distinct biological subtypes or to determine whether recurrence reflects immune memory, persistent local inflammation, ongoing antigen exposure, or delayed tissue recovery. Nevertheless, the observed patterns of steroid dependence, relapse during tapering, and recurrence after rechallenge support close follow-up and individualized decisions regarding ICI resumption (Li et al., 2023).

### 4.4 Outcome definition and phenotype-driven signal dilution

The most important pharmacovigilance finding of this study is that signal strength depended strongly on outcome definition. This issue is particularly relevant for urinary tract toxicity because “cystitis” is a descriptive term rather than an etiologic diagnosis. In routine oncology practice and in spontaneous-reporting databases, cystitis may reflect bacterial infection, radiation-related injury, tumor involvement, catheter irritation, drug-induced inflammation, or immune-mediated urothelial injury. Aggregating these biologically distinct conditions under a single endpoint can reduce etiologic coherence and dilute a true reporting signal.

This effect was evident in our FAERS analysis. The broad definition, which included the nonspecific Preferred Term “cystitis”, identified 397 ICI-associated cases but produced only a weak overall signal (ROR, 1.12; 95% CI, 1.02–1.24). In contrast, the high-specificity definition, restricted to IMMUNE-MEDIATED CYSTITIS, CYSTITIS NONINFECTIVE, and URETERITIS, identified 151 cases and yielded a substantially stronger signal (ROR, 9.04; 95% CI, 7.63–10.70). This difference is unlikely to indicate a biological difference between two separate patient populations. Rather, it reflects the influence of endpoint construction on pharmacovigilance signal detection.

We therefore interpret the high-specificity endpoint as phenotype-enriched rather than immune-confirmed. *Nevertheless, the inclusion of etiologically annotated Preferred Terms such as IMMUNE-MEDIATED CYSTITIS may introduce reporter-attribution bias, because coding decisions may already incorporate clinical suspicion of immune causality.* However, the signal was not driven by this term alone; the high-specificity set also included “cystitis noninfective” and “ureteritis”, and most CASEIDs did not simply represent overlapping coding of multiple high-specificity terms. In addition, the signal persisted after exclusion of cases with co-reported urinary infection-related Preferred Terms. These observations support the view that high-specificity MedDRA construction captured a more coherent urothelial inflammatory phenotype, while not proving causality in individual reports.

### 4.5 Clinical and methodological implications

The present study should be interpreted in relation to two recent developments in this field. The Wang cohort provided a detailed clinical description of ICI-related ureteritis and cystitis, particularly upper urinary tract involvement and relapse during corticosteroid tapering. The pharmacovigilance study by Frey highlighted the detectability of immune-mediated cystitis in FAERS when a highly specific MedDRA term was used. These studies addressed different parts of the same problem: one emphasized clinical phenotype, the other database signal detection. The current analysis attempted to connect these two levels by using the published clinical phenotype to inform FAERS endpoint construction.

Neither evidence source is sufficient on its own. Published cases provide detailed clinical, radiological, pathological, and treatment-response information, but they are vulnerable to selective reporting and cannot define the population-level reporting pattern. FAERS provides scale and signal-detection capacity, but it lacks denominator data, standardized diagnostic confirmation, and sufficient clinical detail to adjudicate causality in individual reports. *Similar pharmacovigilance approaches have also been applied to other emerging immunotherapies, including CAR-T cell therapy and intravesical Bacillus Calmette–Guérin, demonstrating the utility of spontaneous reporting systems for identifying safety patterns of rare treatment-related events* (Peng et al., 2025; Peng et al., 2024). *Furthermore, class-level estimates should be interpreted cautiously because some FAERS CASEIDs involved sequential or combination ICI exposure, particularly CTLA-4 plus PD-1 regimens, and therefore do not represent mutually exclusive treatment comparisons.* Therefore, the FAERS findings should be viewed as population-scale pharmacovigilance support for a clinicopathologically recognizable syndrome, not as proof of incidence, absolute risk, or comparative risk between ICI regimens.

Clinically, ICI-associated cystitis and ureteritis should be considered when persistent urinary symptoms occur in temporal association with ICI exposure, particularly when cultures are negative, pyuria or hematuria is sterile, imaging or cystoscopy shows inflammatory change, and symptoms fail to respond as expected to antimicrobial therapy. The diagnosis should remain one of careful attribution. Infection, obstruction, tumor involvement, radiation injury, prior urologic disease, and concomitant drugs must be actively excluded before urinary symptoms are assigned to an immune-related mechanism. *Earlier recognition of this phenotype may reduce unnecessary antimicrobial exposure and allow timely consideration of immunosuppressive therapy in appropriately selected patients.*

Several limitations deserve emphasis. Published cases may overrepresent severe, unusual, steroid-responsive, or biopsy-confirmed presentations, whereas mild or self-limited cases may never be reported. Because the literature component was designed as a structured pooled case review rather than a formal systematic review, publication bias cannot be excluded. *The high proportion of serious outcomes among high-specificity FAERS cases may also reflect selective reporting of clinically recognized or hospitalized cases, particularly when etiologically annotated terms are used. Therefore, these findings should not be interpreted as indicating that urinary irAEs are intrinsically severe. In addition, spontaneous reporting databases are influenced by under-reporting, stimulated reporting after recognition of rare toxicities, differences in reporting intensity among individual ICIs, and confounding related to underlying malignancies, urinary comorbidities, and concomitant anticancer therapies.* Class-level and agent-level analyses were also not mutually exclusive because a single CASEID could include sequential or combination ICI exposure. TTO also requires caution: the primary TTO estimate came from published cases, whereas FAERS-based TTO was evaluable in only 29 of 151 high-specificity ICI-associated cases and was therefore treated as exploratory. *The difference between published-case and FAERS-based TTO likely reflects differences in case ascertainment and reporting completeness rather than distinct biological onset patterns.*

Despite these limitations, the evidence is internally consistent. Published cases show a recurring pattern of sterile urinary symptoms, inflammatory urinary tract findings, lymphocyte-predominant pathology in biopsied cases, corticosteroid responsiveness, and recurrence during steroid tapering or after rechallenge. FAERS showed a robust disproportionality signal only when the endpoint was restricted to clinically coherent high-specificity terms. Together, these findings support ICI-associated cystitis and ureteritis as an uncommon but clinically meaningful urothelial irAE spectrum.

## 5 Conclusion

ICI-associated cystitis and ureteritis represent an uncommon but clinically important urothelial immune-related adverse event spectrum. Published cases support a recognizable phenotype characterized by persistent sterile urinary symptoms, inflammatory imaging or cystoscopic abnormalities, occasional upper urinary tract involvement, response to immunosuppression, and risk of recurrence during corticosteroid tapering or after ICI rechallenge. In FAERS, the broad cystitis-inclusive definition produced only a weak overall signal, whereas a high-specificity, phenotype-aligned definition revealed a strong and internally consistent disproportionality signal at the overall ICI level and in class-level analyses. These findings highlight the importance of phenotype-aware outcome construction for detecting clinically coherent safety signals. Clinically, persistent cystitis- or ureteritis-like symptoms during ICI therapy should prompt evaluation for immune-mediated urinary tract toxicity after infection, malignancy, obstruction, radiation injury, and other competing etiologies have been excluded.

## Data Availability

The original contributions presented in the study are included in the article/Supplementary Material, further inquiries can be directed to the corresponding author.

## References

[B1] Alhusari L. Abdallah M. Nwanwene K. Shenouda M. (2024). Acute non-infectious cystitis secondary to immune-related adverse events in a patient receiving pembrolizumab for treatment of non-small cell lung cancer: a case report. Cureus 16 (3), e55666. 10.7759/cureus.55666 PMC10997305 38586668

[B2] Anraku T. Hashidate H. Imai T. Kawakami Y. (2023). Successful treatment of immune-related cystitis with bladder hydrodistension. IJU Case Rep. 6 (4), 211–215. 10.1002/iju5.12588 PMC10315250 37405028

[B3] Bate A. Evans S. J. (2009). Quantitative signal detection using spontaneous ADR reporting. Pharmacoepidemiol. Drug Safety 18 (6), 427–436. 10.1002/pds.1742 19358225

[B4] Brahmer J. R. Abu-Sbeih H. Ascierto P. A. Brufsky J. Cappelli L. C. Cortazar F. B. (2021). Society for immunotherapy of cancer (SITC) clinical practice guideline on immune checkpoint inhibitor-related adverse events. J. Immunotherapy Cancer 9 (6), e2435. 10.1136/jitc-2021-002435 PMC8237720 34172516

[B5] Di C. Yu T. Ni L. (2024). Non-bacterial cystitis caused by pembrolizumab therapy for adenocarcinoma of the lung: a case report. Front. Immunol. 15, 1423123. 10.3389/fimmu.2024.1423123 PMC11257856 39034999

[B6] El Husseini K. Lafoeste H. Mansuet-Lupo A. Arrondeau J. Villeminey C. Bennani S. (2021). A case of severe interstitial cystitis associated with pembrolizumab. Curr. Problems Cancer Case Rep. 4, 100101. 10.1016/j.cpccr.2021.100101

[B7] Fan Y. Zhao J. Mi Y. Zhang Z. Geng Y. Zhou L. (2023). Recurrent cystitis associated with 2 programmed death 1 inhibitors. J. Immunother. 46 (9), 341–345. 10.1097/cji.0000000000000484 37721343 PMC10540753

[B8] Frey C. (2026). Association between immune-mediated cystitis and PD-1, PD-L1, CTLA-4, and LAG-3 immune checkpoint inhibitors: a pharmacovigilance study from the FAERS database. Urologia 93 (3), 396-401. 10.1177/03915603261446420 42059249

[B9] Fukunaga H. Sumii K. Kawamura S. Okuno M. Taguchi I. Kawabata G. (2022). A case of steroid-resistant cystitis as an immune-related adverse event during treatment with nivolumab for lung cancer, which was successfully treated with infliximab. IJU Case Rep. 5 (6), 521–523. 10.1002/iju5.12532 36341187 PMC9626353

[B10] Fusaroli M. Salvo F. Begaud B. AlShammari T. M. Bate A. Battini V. (2024a). The reporting of a disproportionality analysis for drug safety signal detection using individual case safety reports in PharmacoVigilance (READUS-PV): development and statement. Drug Saf. 47 (6), 575–584. 10.1007/s40264-024-01421-9 PMC11116242 38713346

[B11] Fusaroli M. Salvo F. Begaud B. AlShammari T. M. Bate A. Battini V. (2024b). The REporting of A disproportionality analysis for DrUg safety signal detection using individual case safety reports in PharmacoVigilance (READUS-PV): explanation and elaboration. Drug Saf. 47 (6), 585–599. 10.1007/s40264-024-01423-7 38713347 PMC11116264

[B12] Haanen J. Obeid M. Spain L. Carbonnel F. Wang Y. Robert C. (2022). Management of toxicities from immunotherapy: ESMO clinical practice guideline for diagnosis, treatment and follow-up. Ann. Oncol. 33 (12), 1217–1238. 10.1016/j.annonc.2022.10.001 36270461

[B13] He X. Tu R. Zeng S. He Z. Liu S. Fang Y. (2022). Non-bacterial cystitis secondary to pembrolizumab: a case report and review of the literature. Curr. Probl. Cancer 46 (4), 100863. 10.1016/j.currproblcancer.2022.100863 35687965

[B14] Inoue Y. Inui N. (2026). Associations between immune-related adverse events and prognosis in cancer patients receiving immune checkpoint inhibitor therapy. Intern. Med. 65 (1), 71–87. 10.2169/internalmedicine.4654-24 39566979 PMC12854964

[B15] Ji J. Lai C. Zhang X. Hu H. (2024). Immune-related adverse events with renal colic as the main manifestation: a case report of sintilimab-induced ureteritis/cystitis treated by ureteral stent and review of the literature. Front. Immunol. 15, 1501415. 10.3389/fimmu.2024.1501415 39763683 PMC11700999

[B16] Li J. Lin Y. (2026). A case report of breast cancer recurrence with cystitis: the impact of immune checkpoint inhibitor therapy on the incidence of cystitis. Immunotherapy 18, 1–6. 10.1080/1750743x.2026.2620589 41601147 PMC13060044

[B17] Li J. Yu Y. Qi X. Du Y. Li C. Q. (2022). Immune-related ureteritis and cystitis induced by immune checkpoint inhibitors: case report and literature review. Front. Immunol. 13, 1051577. 10.3389/fimmu.2022.1051577 36685488 PMC9853439

[B18] Li S. Zheng K. Xu Y. Wang M. (2023). Immune checkpoint inhibitors related cystoureteritis: a case report and literature review. Zhongguo Fei Ai Za Zhi 26 (9), 709–716. 10.3779/j.issn.1009-3419.2023.106.17 37985157 PMC10600747

[B19] Li X. Ao M. Tian S. Chen Y. Chen H. Ta N. (2025). Prolonged survival of thoracic SMARCA4-deficient undifferentiated tumor with immune-related cystitis: a case report and literature review. Ther. Adv. Respir. Dis. 19, 1591002279. 10.1177/17534666251385689 PMC12559638 41126608

[B20] Liu Y. Song Y. Wu J. Li J. Yang W. Du Y. (2026). A systematic review and network Meta analysis of first-line immunotherapy for advanced urothelial carcinoma. Cancer Drug Resist 9, 21. 10.20517/cdr.2026.22 42433711 PMC13352428

[B21] Ni C. Zhao Y. Qian H. Fu H. Yan Y. Y. Qiu Y. S. (2023). Long survival in a pancreatic carcinoma patient with multi-organ toxicities after sintilimab treatment: a case report. Front. Pharmacol. 14, 1121122. 10.3389/fphar.2023.1121122 36744247 PMC9894891

[B22] Obayashi A. Hamada-Nishimoto M. Fujimoto Y. Yoshimoto Y. Takahara S. (2022). Non-bacterial cystitis with increased expression of programmed cell death ligand 1 in the urothelium: an unusual immune-related adverse event after atezolizumab administration for metastatic breast cancer. Cureus 14 (5), e25486. 10.7759/cureus.25486 35800819 PMC9246443

[B23] Ozaki K. Takahashi H. Murakami Y. Kiyoku H. Kanayama H. (2017). A case of cystitis after administration of nivolumab. Int. Cancer Conf. J. 6 (4), 164–166. 10.1007/s13691-017-0298-6 31149494 PMC6498356

[B24] Peng Y. Song Y. Qin C. Du Y. Xu T. (2024). Safety profiles of intravesical bacillus calmette-guerin in bladder cancer. Hum. Vaccin Immunother. 20 (1), 2425529. 10.1080/21645515.2024.2425529 39539079 PMC11572315

[B25] Peng Y. Song Y. Lin J. Qin C. Du Y. Xu T. (2025). Pharmacovigilance analysis of secondary primary malignancies and antibiotic interactions in CAR-T cell therapies. Ther. Adv. Drug Saf. 16, 1601793090. 10.1177/20420986251340866 40485666 PMC12144370

[B26] Peng Z. Yang J. Li H. Lei W. Yang H. Liu X. (2026). Case report: successful rechallenge of cadonilimab-induced cystitis, adrenal insufficiency, and diabetic ketoacidosis in a patient with metastatic gastric-type endocervical adenocarcinoma. Front. Oncology 16, 1836563. 10.3389/fonc.2026.1836563 PMC13229985 42245700

[B27] Schneider B. J. Naidoo J. Santomasso B. D. Lacchetti C. Adkins S. Anadkat M. (2021). Management of immune-related adverse events in patients treated with immune checkpoint inhibitor therapy: ASCO guideline update. J. Clin. Oncol. 39 (36), 4073–4126. 10.1200/JCO.21.01440 34724392

[B28] Schotland P. Racz R. Jackson D. Levin R. Strauss D. G. Burkhart K. (2018). Target-adverse event profiles to augment pharmacovigilance: a pilot study with six new molecular entities. CPT Pharmacometrics Syst. Pharmacol. 7 (12), 809–817. 10.1002/psp4.12356 30354029 PMC6310867

[B29] Shimatani K. Yoshimoto T. Doi Y. Sonoda T. Yamamoto S. Kanematsu A. (2018). Two cases of nonbacterial cystitis associated with nivolumab, the anti-programmed-death-receptor-1 inhibitor. Urol. Case Rep. 17, 97–99. 10.1016/j.eucr.2017.12.006 29541592 PMC5849865

[B30] Song Y. Jiang S. Peng Y. Qin C. Du Y. Xu T. (2024). Effect of FGFR alteration on prognosis in 1963 urothelial carcinoma patients with immune checkpoint inhibitors: implying combination of FGFR inhibitor and immunotherapy for FGFR-altered urothelial carcinoma. Pharmacol. Res. 205, 107230. 10.1016/j.phrs.2024.107230 38788820

[B31] Song Y. Du Y. Jiang S. Peng Y. Luo X. Xu T. (2025). Efficacy and safety of selective pan-fibroblast growth factor receptor (FGFR) tyrosine kinase inhibitors in FGFR-altered urothelial carcinoma. Pharmacol. Res. 211, 107543. 10.1016/j.phrs.2024.107543 39662649

[B32] Thompson J. A. Schneider B. J. Brahmer J. Zaid M. A. Achufusi A. Armand P. (2024). NCCN guidelines® insights: management of immunotherapy-related toxicities, version 2.2024: featured updates to the NCCN guidelines. J. Natl. Compr. Cancer Netw. 22 (9), 582–592. 10.6004/jnccn.2024.0057 39536465

[B33] Tu L. Ye Y. Tang X. Liang Z. You Q. Zhou J. (2021). Case report: a case of Sintilimab-Induced cystitis/ureteritis and review of Sintilimab-Related adverse events. Front. Oncol. 11, 757069. 10.3389/fonc.2021.757069 35004277 PMC8733470

[B34] Ueki Y. Matsuki M. Kubo T. Morita R. Hirohashi Y. Sato S. (2020). Non-bacterial cystitis with increased expression of programmed death-ligand 1 in the urothelium: an unusual immune-related adverse event during treatment with pembrolizumab for lung adenocarcinoma. IJU Case Rep. 3 (6), 266–269. 10.1002/iju5.12211 33163921 PMC7609190

[B35] Wang J. Yang T. Xu J. (2020). Therapeutic development of immune checkpoint inhibitors. Adv. Exp. Med. Biol. 1248, 619–649. 10.1007/978-981-15-3266-5_23 32185726

[B36] Wang Z. Zhu L. Huang Y. Peng L. (2023). Successful treatment of immune-related cystitis by chai-ling-tang (Sairei-To) in a gastric carcinoma patient: case report and literature review. Explore (NY) 19 (3), 458–462. 10.1016/j.explore.2022.04.002 35469747

[B37] Wang C. Nie M. Liu Y. Qiu W. Zhang Z. Zhou N. (2025). Single-center experience with immune checkpoint inhibitor-related ureteritis and cystitis. Front. Immunol. 16, 1727822. 10.3389/fimmu.2025.1727822 41641271 PMC12864438

[B38] Yajima S. Nakanishi Y. Matsumoto S. Tanabe K. Sugano M. Masuda H. (2021). Improvement of urinary symptoms after bladder biopsy: a case of pathologically proven allergy-related cystitis during administration of nivolumab. IJU Case Rep. 4 (4), 213–215. 10.1002/iju5.12286 34308272 PMC8294138

[B39] Yan Y. Dou Q. Wu X. Zhang X. Liu X. Si X. (2026). Immune checkpoint inhibitor-induced ureteritis and cystitis in patients with lung cancer and uterine malignancies: a case series and literature review. J. Immunother. 49 (3), 108-114. 10.1097/CJI.0000000000000581 41036598 PMC12978721

[B40] Yin Q. Wu L. Han L. Zheng X. Tong R. Li L. (2023). Immune-related adverse events of immune checkpoint inhibitors: a review. Front. Immunology 14, 1167975. 10.3389/fimmu.2023.1167975 PMC10247998 37304306

[B41] Yuan Y. Lou L. Zhu S. Yan D. (2025). Immunotherapy-related cystitis induced by nivolumab: a case report and review of the literature. Urol. Case Rep. 62, 103132. 10.1016/j.eucr.2025.103132 PMC12310066 40741426

[B42] Zhang P. Yin C. Yang M. (2024). Case reports of immune-related cystitis and the antibody combination hypothesis. Immunotherapy 16 (16-17), 1039–1047. 10.1080/1750743X.2024.2389761 39263930 PMC11492643

[B43] Zhou Q. Qin Z. Yan P. Wang Q. Qu J. Chen Y. (2023). Immune-related adverse events with severe pain and ureteral expansion as the main manifestations: a case report of tislelizumab-induced ureteritis/cystitis and review of the literature. Front. Immunol. 14, 1226993. 10.3389/fimmu.2023.1226993 37869004 PMC10587548

[B44] Zhu L. Wang Z. Stebbing J. Peng L. (2021a). Immunotherapy-related cystitis: case report and review of the literature. Onco Targets Ther. 14, 4321–4328. 10.2147/ott.s321965 PMC8336986 34366676

[B45] Zhu S. Bian L. Lv J. Liu B. Shen J. (2021b). A case report of non-bacterial cystitis caused by immune checkpoint inhibitors. Front. Immunol. 12, 788629. 10.3389/fimmu.2021.788629 PMC8733335 35003107

